# lncRNA_Mdeep: An Alignment-Free Predictor for Distinguishing Long Non-Coding RNAs from Protein-Coding Transcripts by Multimodal Deep Learning

**DOI:** 10.3390/ijms21155222

**Published:** 2020-07-23

**Authors:** Xiao-Nan Fan, Shao-Wu Zhang, Song-Yao Zhang, Jin-Jie Ni

**Affiliations:** Key Laboratory of Information Fusion Technology of Ministry of Education, School of Automation, Northwestern Polytechnical University, Xi’an 710072, China; fanxn@nwpu.edu.cn (X.-N.F.); zsy@nwpu.edu.cn (S.-Y.Z.); EMINEM_NI@foxmail.com (J.-J.N.)

**Keywords:** long noncoding RNA, alignment-free, multimodal learning, deep learning

## Abstract

Long non-coding RNAs (lncRNAs) play crucial roles in diverse biological processes and human complex diseases. Distinguishing lncRNAs from protein-coding transcripts is a fundamental step for analyzing the lncRNA functional mechanism. However, the experimental identification of lncRNAs is expensive and time-consuming. In this study, we presented an alignment-free multimodal deep learning framework (namely lncRNA_Mdeep) to distinguish lncRNAs from protein-coding transcripts. LncRNA_Mdeep incorporated three different input modalities, then a multimodal deep learning framework was built for learning the high-level abstract representations and predicting the probability whether a transcript was lncRNA or not. LncRNA_Mdeep achieved 98.73% prediction accuracy in a 10-fold cross-validation test on humans. Compared with other eight state-of-the-art methods, lncRNA_Mdeep showed 93.12% prediction accuracy independent test on humans, which was 0.94%~15.41% higher than that of other eight methods. In addition, the results on 11 cross-species datasets showed that lncRNA_Mdeep was a powerful predictor for predicting lncRNAs.

## 1. Introduction

Long non-coding RNA (lncRNAs) are defined as non-protein-coding transcripts with a length of more than 200 nucleotides. Several studies reveal that more than 70% of the human genome is capable of being transcribed, whereas less than 2% of the genome can be translated into proteins [1]. LncRNAs make up the largest portion of the non-protein-coding transcripts [2,3,4] and show critical roles in cellular function, development, and diseases [5,6,7].

LncRNA identification is the fundamental step of lncRNA-related researches, which has drawn a lot of attention in recent years. Several computational methods are developed for distinguishing lncRNAs from protein-coding transcripts. Existing computation methods can be mainly categorized into alignment-based methods [8,9,10,11,12,13] and alignment-free methods [14,15,16,17,18,19,20,21]. The alignment-based methods generally align the transcripts against a comprehensive reference protein database for predicting lncRNAs; for example, CPC (Coding Potential Calculator) [8] aligns transcripts against UniRef90 dataset [22] using BLSATX [23] tool; lncRNA-ID [11] and lncADeep [13] align the transcripts against Pfam dataset [24] using HMMER [25] tool. This kind of method heavily relies on the quality of alignments, which will be influenced by the performance of multiple-sequence alignment tools and the quality of reference databases. Furthermore, the alignment process is extremely time-consuming [15,21]. To avoid the drawback caused by alignment, the alignment-free methods are developed to distinguish lncRNAs from protein-coding transcripts. Without considering conservation features, CNCI (Coding-Non-Coding Index) [14] extracts five features (i.e., the length and S-score of most-like coding domain sequence, length-percentage, score-distance, and codon-bias) by profiling adjoining nucleotide triplets to represent the transcript sequences. CPAT (Coding-potential Assessment Tool) [15] calculates open reading frame size, open reading frame coverage, Fickett TESTCODE score, and hexamer score. PLEK (Predictor of LncRNA and mEssenger RNAs based on an improved *k*-mer scheme) [16] proposes an improved *k*-mer feature. These methods adopt different machine learning algorithms to build the classifiers for predicting lncRNAs. For example, CNCI and PLEK use a support vector machine (SVM), and CPAT uses logistic regression. Except for these conventional machine learning algorithms, deep learning, a branch of machine learning, has been applied for lncRNA identification. For example, lncRNA-MFDL (identification of lncRNA by fusing Multiple Features and using Deep Learning) [17] predicts lncRNAs by fusing multiple features and a deep stacking network, and Tripathi et al. [18] proposed the DeepLNC method to predict lncRNAs by *k*-mer features and a deep neural network classifier. Although these two methods based on deep learning algorithms achieve a better performance than previous conventional machine learning algorithms to predict lncRNAs, they still depend on manually crafted features and fail to learn intrinsic features automatically from raw transcript sequences. Recently, a deep learning-based method, lncRNAnet [20], has been proposed to predict lncRNAs. LncRNAnet builds a convolutional neural network (CNN) for detecting the open reading frame (ORF) indicator and a recurrent neural network (RNN) for modeling RNA sequence to predict lncRNAs, not taking into consideration at all the manually crafted features.

In this study, we proposed an alignment-free method, lncRNA_Mdeep, to distinguish lncRNAs from protein-coding transcripts by using multimodal deep learning. The novelties of lncRNA_Mdeep mainly included: (1) LncRNA_Mdeep successfully integrated manually crafted features and raw transcript sequences; (2) LncRNA_Mdeep effectively extracted high-level abstract representations from multiple deep learning models based on different raw input features; (3) LncRNA_Mdeep successfully distinguished lncRNAs from protein-coding transcripts in not only human dataset but also multiple cross-species datasets. To validate our lncRNA_Mdeep, we tested it on a human dataset containing 46,000 transcripts in 10-fold cross-validation (10CV) test and compared it with the other seven model architectures. Furthermore, we compared lncRNA_Mdeep with other eight state-of-the-art methods on the human and 11 cross-species datasets in an independent test. The results showed that lncRNA_Mdeep could effectively distinguish lncRNAs from protein-coding transcripts.

## 2. Results

**Figure 1:**
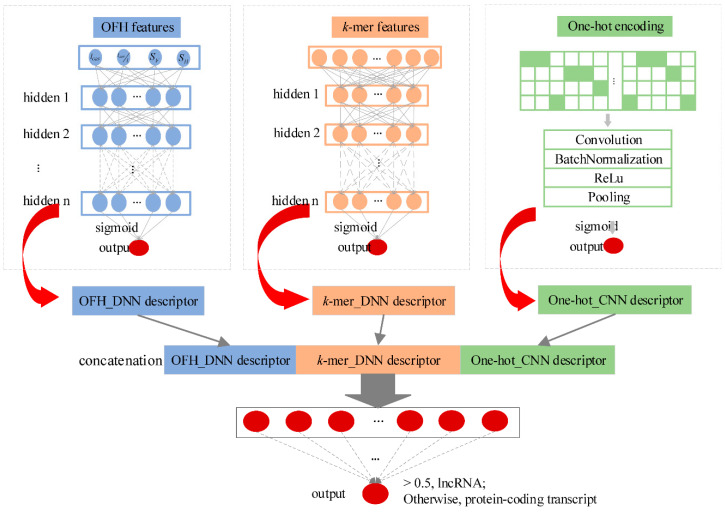
Overview of the lncRNA_Mdeep.

We developed an alignment-free multimodal deep learning framework (namely lncRNA_Mdeep) to distinguish lncRNAs from protein-coding transcripts (Figure 1, Materials and Methods). LncRNA_Mdeep first extracted the OFH (the length and coverage of ORF, Fickett score, and Hexamer score) feature and *k*-mer feature from transcript sequences and used a one-hot encoding strategy to encode the transcript sequences, then two deep neural network (DNN) models and a CNN model were built to mine the high-level representations. Finally, the learned representations were fused, and a multimodal deep learning framework was built to distinguish lncRNAs from protein-coding transcripts.

To evaluate the performance of lncRNA_Mdeep, we first investigated the performance of lncRNA_Mdeep with different model architectures on a human dataset in 10CV test and showed the effect of different hyper-parameters in DNNs and CNN, then compared lncRNA_Mdeep with eight existing state-of-the-art methods (i.e., CNCI [14], CPAT [15], PLEK [16], lncRNA-MEDL [17], CPC2 [19], lncRNAnet [20], LncFinder^1^, and LncFinder^2^ [21]) on human and 11 cross-species datasets in an independent test. LncFinder^1^ means the LncFinder without the secondary structure, and LncFinder^2^ means LncFinder with the secondary structure. The overall performance was measured by accuracy (ACC), sensitivity (S*_n_*), specificity (S*_p_*), and Matthew’s correlation coefficient (MCC).

LncRNA_Mdeep was implemented in Python 3 using Keras 2.2.4 [26] with the backend of Tensorflow-gpu (1.9.0) [27]. All the experiments were implemented on an Ubuntu system with an NVIDIA TITAN V GV100.

### 2.1. Performance of lncRNA_Mdeep

#### 2.1.1. Performance of Different Model Architectures

We separately implemented the DNN model with the OFH feature as input (namely OFH_DNN), DNN model with the *k*-mer feature as input (namely *k*-mer_DNN), CNN model with one-hot encoding as input (namely One-hot_CNN), the combinations of these models (i.e., OFH_DNN + *k*-mer_DNN, *k*-mer_DNN + One-hot_CNN, and OFH_DNN + One-hot_CNN), and the decision fusion of three models in 10CV test. The results are shown in Table 1, from which we could see that the accuracy, *S_n_*, *S_p_*, and MCC of lncRNA_Mdeep were 98.73%, 98.95%, 98.52%, and 0.9748, respectively. By comparing the performance of OFH_DNN, *k*-mer_DNN, One-hot_CNN, and lncRNA_Mdeep, we found that the accuracy of lncRNA_Mdeep was over 2.20% higher than that of OFH_DNN, *k*-mer_DNN, and One-hot_CNN. The MCC of lncRNA_Mdeep was over 0.0441 higher than that of OFH_DNN, *k*-mer_DNN, and One-hot_CNN. The *S_n_* of lncRNA_Mdeep was over 1.94% higher than that of OFH_DNN, *k*-mer_DNN, and One-hot_CNN. The *S_p_* of lncRNA_Mdeep was over 1.48% higher than that of OFH_DNN, *k*-mer_DNN, and One-hot_CNN. These results showed that lncRNA_Mdeep through incorporating three different input modalities achieved better performance than individual models. In addition, *k*-mer_DNN showed the best performance among three individual models (i.e., OFH_DNN, *k*-mer_DNN, and One-hot_CNN).

By comparing the performance of the different combinations of three individual models and lncRNA_Mdeep, we found that the accuracy of lncRNA_Mdeep was 2.76%, 0.37%, and 1.13% higher than that of OFH_DNN + *k*-mer_DNN, *k*-mer_DNN + One-hot_CNN, and OFH_DNN + One-hot_CNN, respectively. The MCC of lncRNA_Mdeep was 0.0537, 0.0074, and 0.0222 higher than that of OFH_DNN + *k*-mer_DNN, *k*-mer_DNN + One-hot_CNN, and OFH_DNN + One-hot_CNN, respectively. These results showed that lncRNA_Mdeep through fusing three models achieved better performance than that of fusing any two models.

Furthermore, we also compared lncRNA_Mdeep with a decision fusion strategy of voting. As shown in Table 1, the performance of lncRNA_Mdeep was 0.31% and 0.0059 higher than that of the voting fusion strategy in terms of accuracy and MCC. To evaluate whether the improvements of lncRNA_Mdeep and other model architectures were significant or not, we calculated the *p*-values between the predicted results of lncRNA_Mdeep and other model architectures using the McNemar’s test [28]. The *p*-values are shown in Supplementary Table S1. All those results showed that lncRNA_Mdeep was a superior deep learning framework, and it could effectively distinguish lncRNAs from protein-coding transcripts. 

#### 2.1.2. Effects of Different Hyper-Parameters

**Figure 2:**
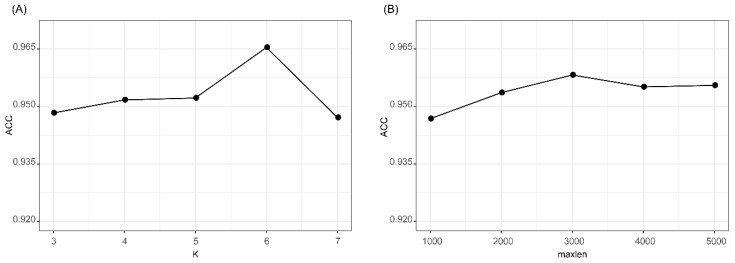
Results of *k*-mer_DNN and One-hot_CNN with different parameters. (**A**) Accuracy of *k*-mer_DNN with different *k* values. (**B**) Accuracy of One-hot_CNN with different *maxlen* values. *k*-mer_DNN, the DNN model with the *k*-mer feature as input. One-hot_CNN, the CNN model with one-hot encoding as input.

We evaluated the effects of two parameters of *k* in *k*-mer feature and *maxlen* for padding one-hot encoding. The accuracies of *k*-mer_DNN and One-hot_CNN in the 10CV test at different *k* and *maxlen* are shown in Figure 2. As shown in Figure 2A, we found that *k*-mer_DNN achieved the highest accuracy when *k* = 6. Results in Figure 2B shows that One-hot_CNN achieved the highest accuracy when *maxlen* = 3000. Therefore, we set *k* = 6, when we extracted the *k*-mer feature, and fixed the one-hot encoding of a transcript as a 4 × *3000* matrix. The results (Supplementary Table S2) of the McNemar’s test for the comparison of different *k* and *maxlen* values showed that the performances of *k*-mer_DNN and One-hot_CNN with selected best *k* and *maxlen* were significantly different from other candidate parameter values. All other hyper-parameters in lncRNA_Mdeep were selected by using a hyperopt [29] strategy, and all parameters were optimized in a searching range, and the best value was selected. All the searching range and best value of hyperparameters are shown in Supplementary Table S3 and Supplementary Figure S1. 

### 2.2. Comparison with Other Existing Methods

We compared lncRNA_Mdeep with the other eight existing alignment-free methods (i.e., CNCI, CPAT, PLEK, lncRNA-MEDL, CPC2, lncRNAnet, LncFinder^1^, and LncFinder^2^) on human datasets and cross-species datasets in an independent test. LncRNA_Mdeep was trained on the human dataset, and since most existing methods do not provide the retraining option, we used their pre-trained models.

#### 2.2.1. Comparison Performance on Human Dataset

We first compared the performance of lncRNA_Mdeep and the other eight existing methods on the human testing dataset. The results are shown in Table 2, from which we could see that lncRNA_Mdeep achieved an accuracy of 93.12%, which was 6.72%, 5.14%, 15.41%, 7.65%, 15.14%, 0.94%, 6.90%, and 6.24% higher than that of CNCI, CPAT, PLEK, lncRNA-MEDL, CPC2, lncRNAnet, LncFinder^1^, and LncFinder^2^, respectively. MCC and *S_p_* of lncRNA_Mdeep were 0.8653 and 88.97%, which were at least 0.0183 and 1.24% higher than that of eight methods. Although CNCI achieved 97.42% sensitivity, which was 0.15% higher than that of lncRNA_Mdeep, it showed lower performance in terms of accuracy, *S_p_*, and MCC. All the improvements of lncRNA_Mdeep were tested by McNemar’s test, and the results showed they were significant. The *p*-values of McNemar’s test are listed in Supplementary Table S4.

#### 2.2.2. Comparison Performance on Cross-Species Datasets

We also compared the performance of lncRNA_Mdeep and other eight existing methods by using 11 cross-species datasets as the independent testing datasets. The results are shown in Table 3. On mouse testing dataset, lncRNA_Mdeep achieved 92.52% accuracy, which was 5.43%, 2.05%, 20.63%, 3.99%, 12.09%, 0.71%, 4.05%, and 3.53% higher than that of CNCI, CPAT, PLEK, lncRNA-MEDL, CPC2, lncRNAnet, LncFinder^1^, and LncFinder^2^, respectively. On other 10 cross-species testing datasets, the accuracies of lncRNA_Mdeep for *Arabidopsis*, *Bos taurus*, *Caenorhabditis elegans*, chicken, chimpanzee, frog, fruit fly, gorilla, pig, and zebrafish were 95.73%, 97.33%, 98.87%, 96.06%, 96.76%, 96.80%, 96.10%, 96.65%, 96.87%, and 96.76%, respectively. LncRNA_Mdeep showed the best performance on 5 out of 11 cross-species testing datasets, lncRNA-MDFL showed the best performance on three cross-species testing datasets, lncFinder^2^ showed the best performance on two cross-species testing datasets, and CPAT showed the best performance on one cross-species testing datasets. The results on the 11 testing datasets showed that lncRNA_Mdeep had the superior performance for distinguishing lncRNAs from protein-coding transcripts.

## 3. Discussion

LncRNA identification is essential for understanding the function and regulatory mechanism of lncRNA. In recent years, several computational methods are developed for distinguishing lncRNAs from protein-coding transcripts. Most of the existing methods have focused on manually extracting features and directly feeding into a classifier (e.g., support vector machine, logistic regression, and random forest) to predict lncRNAs. These predictors depend on the effectiveness of manually crafted features and fail to automatically learn intrinsic representations from raw transcript sequences. To address this issue, lncRNA_Mdeep is proposed to identify lncRNAs by multimodal deep learning. LncRNA_Mdeep can successfully integrate the manually crafted features and the raw transcript sequences. It also successfully learns high-level abstract representations based on different raw input features and integrates learned high-level abstract representations by a multimodal deep learning model to predict lncRNAs. 

Our experience results showed that lncRNA_Mdeep was a superior predictor for distinguishing lncRNAs from protein-coding transcripts. We compared lncRNA_Mdeep with other different model architectures on the human dataset in the 10CV test and compared lncRNA_Mdeep with existing eight state-of-the-art methods on humans and 11 cross-species datasets in an independent test. The results in Table 1, Table 2 and Table 3 showed that lncRNA_Mdeep was a superior multimodal framework, and it achieved a better performance than other methods on human and 11 cross-species datasets. Furthermore, considering the possible false annotation for manually annotated GENCODE lncRNA transcripts without 5′ cap and 3′ polyA signals, we re-filtered the lncRNA transcripts downloaded from GENOCDE and collected a more high-quality dataset. Our lncRNA_Mdeep still showed a good performance on the high-quality dataset, and that was better than other model architectures on the high-quality dataset (Supplementary Table S5). 

Although lncRNA_Mdeep showed a superior performance to identify lncRNAs, there were still several issues that need to be addressed in the future. First, lncRNA_Mdeep used a one-hot encoding strategy to encode the raw transcript sequence and set up a parameter of *maxlen* to meet the input requirement of the CNN model, but we expected a more effective encoding strategy to encode the transcript sequences with variable-length. Second, the deep learning model was like a black box, which could interpret the meaning of learned high-level abstract representations, but we expected a good way to analyze the learned high-level abstract representations. Third, we expected a more reliable annotation pipeline for lncRNA transcripts without a 5′ cap and 3′ polyA signals to train a computational model to distinguish lncRNA transcripts from protein-coding transcripts.

## 4. Material and Methods

### 4.1. Dataset

Human lncRNA and protein-coding transcripts were downloaded from GENCODE release 30 [30]. After removing the transcripts whose length was less than 200 nt, we obtained 29,698 lncRNAs and 75,153 protein-coding transcripts, from which we randomly selected 23,000 lncRNAs and 23,000 protein-coding transcripts to construct the training dataset. In the remaining transcripts, we randomly selected 6000 lncRNAs and 6000 protein-coding transcripts to construct the testing dataset.

We also built other 11 cross-species testing datasets, in which mouse lncRNAs and protein-coding transcripts were downloaded from GENCODE release M20 [30], and other 10 cross-species (e.g., *Arabidopsis*, chicken, *Bos taurus*, *C. elegans*, chimpanzee, frog, fruit fly, gorilla, pig, and zebrafish) testing datasets were downloaded from RefSeq *v*. 94 [31]. The statistics of all datasets are listed in Supplementary Table S6.

### 4.2. LncRNA_Mdeep

LncRNA_Mdeep mainly consisted of the following phases: (1) Extracted the OFH feature and *k*-mer feature from transcript sequences and used one-hot encoding strategy to encode the transcript sequences; (2) Built two DNN models and a CNN model to mine the high-level representations from OFH feature, *k*-mer feature, and one-hot encoding of transcript sequences, respectively; (3) Fused the learned representations (namely, OFH_DNN descriptor, *k*-mer_DNN descriptor, and One-hot_CNN descriptor) to represent the transcript sequences; (4) Fed three descriptors into a DNN to distinguish lncRNAs from protein-coding transcripts. The overview of lncRNA_Mdeep is illustrated in Figure 1.

#### 4.2.1. Feature Extraction and One-Hot Encoding

Given a transcript sequence $T=N_{1}N_{2}N_{3}N_{4}\cdots N_{L}$ with *L* nucleotides, where $N_{1}$ denotes the first nucleotide, $N_{2}$ denotes the second nucleotide, and so on, we extracted two kinds of features from the transcript sequences to convert them into vectors. 

The first one was the OFH feature, which consisted of the length and coverage of the open reading frame (ORF), a Fickett score, and a Hexamer score. We first calculated the length and the coverage of ORF, which was identified as the longest reading frame in three forward frames starting with a start codon and ending with a stop codon, then obtained the Fickett score *S_F_* from the literature [32]. Fickett score *S_F_* could be attained by calculating the percentage compositions of A, C, G, T and their position values (i.e., A_pos_, C*_pos_*, G*_pos_*, and T*_pos_*) with the formula $B_{pos}=\frac{MAX\left(B_{1},B_{2},B_{3}\right)}{MIN\left(B_{1},B_{2},B_{3}\right)+1}$, where *B*_1_, *B*_2_, *B*_3_ denote the occurrence number of nucleotide B (i.e., A, C, G, and T) in first, second, and third position of the codon, respectively, and transforming these eight values to a TESTCODE score. The Hexamer score *S_H_* was calculated by using the formula $S_{H}=\frac{1}{m}\overset{m}{\underset{i=1}{\sum }}\log\left(\frac{F_{c}\left(h_{i}\right)}{F_{nc}\left(h_{i}\right)}\right),m=L-6+1$, where $F_{c}\left(h_{i}\right)$ and $F_{nc}\left(h_{i}\right)$ (*i* = 1, 2, …, 4096) represent in-frame coding and non-coding hexamer frequency, respectively. Finally, the OFH feature could be represented as XOFH=[lORF,lORFL,SF,SH], where *l_ORF_* denotes the length of ORF, *S_F_* denotes the Fickett score, and *S_H_* denotes the Hexamer score.

The second feature was the *k*-mer frequency feature denoted as $X_{kmer}=\left[f_{1},f_{2},\cdots ,f_{i},\cdots ,f_{4^{k}}\right]$, where $f_{i}$ is the occurrence frequency of *k* neighboring bases in the transcript sequence. 

One-hot encoding translated the A, T, C, G characters into a binary vector of (1,0,0,0), (0,1,0,0), (0,0,1,0) and (0,0,0,1), respectively. Therefore, the transcript with length *L* was denoted as a 4 × *L* matrix, i.e., $X_{Onehot}=\left[x_{1},\cdots ,x_{i},\cdots ,x_{L}\right]$, where $x_{i}$ is the corresponding binary vector of *i^th^* nucleotide in the transcript. 

#### 4.2.2. High-Level Abstract Representations 

Two DNN models and a CNN model were built to learn the hidden high-level abstract representations from different input modalities. DNN model consisted of an input layer, multiple hidden layers, and an output layer, which is used to model high-level abstractions in input data with a deep architecture composed of multiple non-linear transformations [33,34]. We built two DNN models for inputs of the OFH feature and *k*-mer feature, respectively. Then, the OFH_DNN descriptor and *k*-mer_DNN descriptor were obtained from the last hidden layers of two DNNs, which denoted the final representations of OFH feature and *k*-mer feature, respectively.

Furthermore, a CNN model was built to learn the hidden high-level abstract representations from the one-hot encoding of transcript sequences. CNN model consisted of the convolution layer, batch normalization, rectified linear unit, and pooling [35].

(1) $$R=Pool\left(ReLU\left(Batch_{\Gamma ,\beta }\left(conv_{M}\left(X\right)\right)\right)\right)$$

where *R* is the output vector of the convolutional module; *X* is the input vector; Γ, β and M are the parameters of batch normalization and convolution layers. Since the input of CNN required fixed-length input, we set a parameter of *maxlen* to make the one-hot encoding of transcript sequence $X_{Onehot}$ to be a 4 × *maxlen* matrix.

(2) $$X_{Onehot}=\{\begin{array}{c} \left[x_{1},x_{2},\cdots ,x_{maxlen}\right],\;L\geq maxlen\\ \left[x_{1},\cdots ,x_{maxlen},0,\cdots ,0\right],\;L<maxlen \end{array}$$

The output of CNN was defined as the one-hot_CNN descriptor, which denoted the high-level abstract representations of raw transcript sequences.

#### 4.2.3. Multimodal Framework

To distinguish the lncRNAs from protein-coding transcripts, we concatenated the OFH_DNN descriptor, *k*-mer_DNN descriptor, and one-hot_CNN descriptor, then fed them into a DNN to predict the probability of the input transcript sequence to be a lncRNA. There were two steps to train our lncRNA_Mdeep. The first step was training a DNN for the OFH feature, a DNN for *k*-mer feature, and a CNN for one-hot encoding, respectively. The parameters in two DNNs and CNN architectures were trained using the labeled data. The second step was learning the parameters of the DNN for final classification and processing a fine-tuning for renewing all parameters in the whole multimodal framework.

### 4.3. Evaluation Metrics

The following metrics of accuracy (ACC), sensitivity (*S_n_*), specificity (*S_p_*), and Matthew’s correlation coefficient (MCC) were used to measure the performance of lncRNA_Mdeep.

(3) ACC=(TP+TN)(TP+FP+TN+FN)

(4) Sn=TP(TP+FN)

(5) Sp=TN(TN+FP)

(6) MCC=(TP×TN−FP×FN)(TP+FP)(TP+FN)(TN+FP)(TN+FN)

where *TP* and *TN* are the number of correctly predicted lncRNAs and protein-coding transcripts, respectively; *FP* and *FN* are the number of incorrectly predicted lncRNAs and protein-coding transcripts, respectively.

## 5. Conclusions

In this study, we proposed a novel multimodal deep learning method (namely lncRNA_Mdeep) to distinguish lncRNAs from protein-coding transcripts. LncRNA_Mdeep first built three individual deep model architectures to learn the hidden high-level abstract representations from three input modalities (i.e., OFH modality, *k*-mer modality, and sequence modality), and high-level representations were fused to feed into another deep model architecture for predicting lncRNAs. The experimental results showed that lncRNA_Mdeep successfully integrated the manually crafted features (i.e., OFH and *k*-mer features) and the raw transcript sequences by using the multimodal framework, and it achieved higher performance than other state-of-the-art methods on human and other 11 cross-species datasets. These results indicated that lncRNA_Mdeep could contribute to the identification of novel lncRNA transcripts.

## Figures and Tables

**Table 1:** Performance of lncRNA_Mdeep and other model architectures in the 10CV test.

	ACC (%)	*S_n_* (%)	*S_p_* (%)	MCC
OFH_DNN	95.74 ± 1.70	94.44 ± 4.89	97.04 ± 2.15	0.9171 ± 0.0307
*k*-mer_DNN	96.53 ± 0.41	96.40 ± 1.11	96.66 ± 0.78	0.9307 ± 0.0082
One-hot_CNN	95.82 ± 0.33	97.01 ± 0.96	94.63 ± 1.19	0.9169 ± 0.0064
OFH_DNN + *k*-mer_DNN	95.97 ± 2.49	96.87 ± 1.05	95.06 ± 5.71	0.9211 ± 0.0449
k-mer_DNN + One-hot_CNN	98.36 ± 0.16	98.70 ± 0.42	98.03 ± 0.50	0.9674 ± 0.0033
OFH_DNN + One-hot_CNN	97.60 ± 1.26	97.78 ± 1.58	97.43 ± 2.33	0.9526 ± 0.0248
Decision fusion	98.42 ± 1.12	99.24 ± 0.45	97.60 ± 2.59	0.9689 ± 0.0212
lncRNA_Mdeep	98.73 ± 0.41	98.95 ± 0.54	98.52 ± 0.92	0.9748 ± 0.0080

OFH_DNN, the DNN model with the OFH feature as input. *k*-mer_DNN: the DNN model with the *k*-mer feature as input. One-hot_CNN: the CNN model with one-hot encoding as input. ACC, accuracy. *S_n_*, sensitivity. *S_p_*, specificity. MCC, Matthew’s correlation coefficient.

**Table 2:** Performance of lncRNA_Mdeep and other eight methods on humans in an independent test.

Methods	ACC (%)	*S_n_* (%)	*S_p_* (%)	MCC
CNCI	86.40	97.42	75.38	0.7463
CPAT	87.98	95.22	80.73	0.7676
PLEK	77.71	97.22	58.20	0.6019
lncRNA-MFDL	85.47	93.43	77.50	0.7185
CPC2	77.98	94.07	61.90	0.5911
lncRNAnet	92.18	96.63	87.73	0.8470
lncFinder^1^	86.22	95.20	77.23	0.7363
lncFinder^2^	86.88	95.98	77.77	0.7501
lncRNA_Mdeep	93.12	97.27	88.97	0.8653

CNCI, coding-non-coding index [14]. CPAT, coding-potential assessment tool [15]. PLEK, predictor of lncRNA and messenger RNAs based on an improved *k*-mer scheme [16]. lncRNA-MFDL, identification of lncRNA by fusing multiple features and using deep learning [17]. CPC2, coding potential calculator 2 [19]. lncRNAnet, lncRNA identification using deep learning [20]. LncFinder^1^: the LncFinder without the secondary structure; LncFinder^2^: the LncFinder with the secondary structure [21]. lncRNA_Mdeep, our method.

**Table 3:** Accuracy (%) of lncRNA_Mdeep and other eight methods on 11 cross-species datasets.

Species	CNCI	CPAT	PLEK	lncRNA-MFDL	CPC2	lncRNAnet	lncFinder^1^	lncFinder^2^	lncRNA_Mdeep
Mouse	87.09	90.47	71.89	88.53	80.43	91.81	88.47	88.99	**92.52**
*Arabidopsis*	79.86	91.39	66.93	**97.30**	93.36	94.60	92.45	93.77	95.73
*Bos taurus*	92.88	97.13	89.32	95.51	96.10	96.30	97.00	97.03	**97.33**
*C. elegans*	77.72	91.48	45.37	97.97	94.75	97.95	87.46	88.55	**98.87**
Chicken	91.52	**97.04**	83.95	96.87	95.22	95.56	96.82	96.64	96.06
Chimpanzee	89.84	96.18	88.99	94.26	95.48	94.78	96.05	96.21	**96.76**
Frog	90.60	96.40	80.90	96.14	96.34	95.53	96.92	**97.26**	96.80
Fruit fly	92.90	96.02	74.43	**96.49**	94.28	95.21	95.33	95.50	96.10
Gorilla	89.37	94.99	86.75	95.12	94.12	94.31	94.72	94.87	**95.65**
Pig	91.73	96.91	87.34	**96.98**	95.86	95.56	96.88	96.82	96.87
Zebrafish	93.59	97.50	85.07	92.17	96.83	95.77	97.54	**97.78**	96.76

## Supplementary Materials

Supplementary materials can be found at https://www.mdpi.com/1422-0067/21/15/5222/s1, Figure S1: The hyper-parameters in lncRNA_Mdeep; Table S1. The p-value of lncRNA_Mdeep and other model architectures in McNemar’s test; Table S2: The p-values of predicted results on different *k* and *maxlen* in McNemar’s test; Table S3: The searching range and best value for each hyperparameter; Table S4: The p-value of lncRNA_Mdeep and other tools on human and 5 cross-species datasets in McNemar’s test; Table S5: Performance of lncRNA_Mdeep and other model architectures on the high-quality dataset * in 10CV test; Table S6: The statistics of all datasets.

## Abbreviations

lncRNA	Long non-coding RNA
SVM	Support vector machine
CNN	Convolutional neural network
RNN	Recurrent neural network
DNN	Deep neural network
ORF	Open reading frame
ACC	Accuracy
*S_n_*	Sensitivity
*S_p_*	Specificity
MCC	Matthew’s correlation coefficient
