# Supplementary material for: lncRNA_Mdeep: An Alignment-Free Predictor for Distinguishing Long Non-Coding RNAs from Protein-Coding Transcripts by Multimodal Deep Learning
Source: Int J Mol Sci. 2020 Jul 23;21(15):5222. doi: 10.3390/ijms21155222 (PMC7432689; doi:10.3390/ijms21155222)
Supplement: Supplementary file 1 [file ijms-21-05222-s001.pdf]

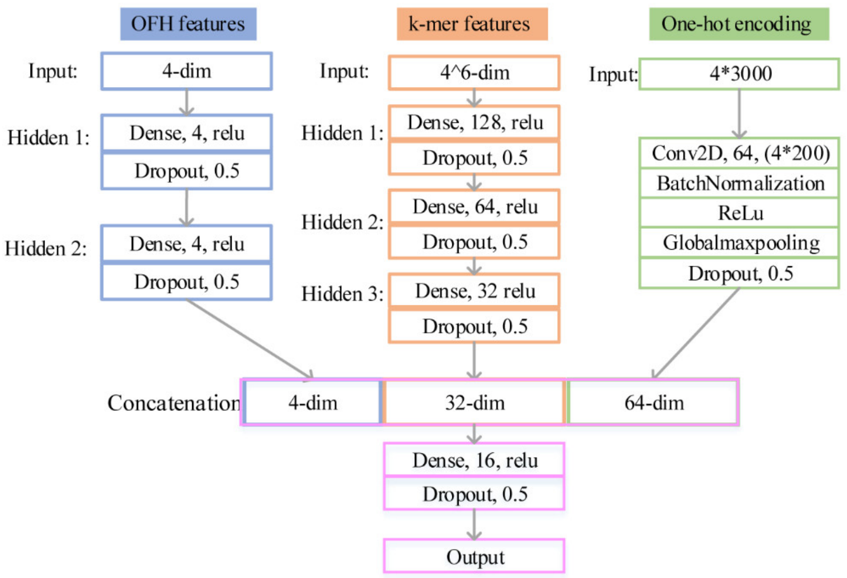

Figure S1. The hyper-parameters in lncRNA_Mdeep

Table S1. The p-value of lncRNA_Mdeep and other model architectures in McNemar's test

	p-value
OFH_DNN v.s. lncRNA_Mdeep	1.3×10^{-188}
k-mer_DNN v.s. lncRNA_Mdeep	3.4×10^{-146}
One-hot_CNN v.s. lncRNA_Mdeep	3.9×10^{-199}
OFH_DNN + k-mer_DNN v.s. lncRNA_Mdeep	5.2×10^{-195}
k-mer_DNN + One-hot_CNN v.s. lncRNA_Mdeep	1.7×10^{-11}
OFH_DNN + One-hot_CNN v.s. lncRNA_Mdeep	2.4×10^{-52}
Decision fusion v.s. lncRNA_Mdeep	9.9×10^{-7}

Table S2. The p-values of predicted results on different *k* and *maxlen* in McNemar's test

k	p-value	*maxlen*	p-value
k = 3 v.s. *k* = 6	1.2×10^{-56}	*maxlen* = 1000 v.s. *maxlen* = 3000	2.3×10^{-32}
k = 4 v.s. *k* = 6	1.2×10^{-56}	*maxlen* = 2000 v.s. *maxlen* = 3000	2.4×10^{-9}
k = 5 v.s. *k* = 6	5.8×10^{-46}	*maxlen* = 4000 v.s. *maxlen* = 3000	4.4×10^{-5}
k = 7 v.s. *k* = 6	5.6×10^{-74}	*maxlen* = 5000 v.s. *maxlen* = 3000	3.3×10^{-4}

Table S3. The searching range and best value for each hyperparameter

	Hyperparameters	Searching range	Best value
OFH_DNN	# Hidden layers	1, 2, 3, 4	2
	# Neurons in hidden layer 1	4, 8, 16	4
	Dropout after hidden layer 1	0.2, 0.3, 0.5	0.5
	# Neurons in hidden layer 2	4, 8, 16	4
	Dropout after hidden layer 2	0.2, 0.3, 0.5	0.5
k-mer_DNN	# Hidden layers	1, 2, 3, 4	3
	# Neurons in hidden layer 1	32, 64, 128, 256	128
	Dropout after hidden layer 1	0.2, 0.3, 0.5	0.5
	# Neurons in hidden layer 2	32, 64, 128, 256	64
	Dropout after hidden layer 2	0.2, 0.3, 0.5	0.5
	# Neurons in hidden layer 3	32, 64, 128, 256	32
	Dropout after hidden layer 3	0.2, 0.3, 0.5	0.5
One-hot_CNN	Kernel size	4×100, 4×200, 4×300	4×200
	# Filters	16, 32, 64	64
	Dropout after Pooling layer	0.2, 0.3, 0.5	0.5
DNN for final classification	# Hidden layers	1, 2, 3, 4	1
	# Neurons in hidden layer	4, 8, 16	16
	Dropout after hidden layer 1	0.2, 0.3, 0.5	0.5

Table S4. The p-value of lncRNA_Mdeep and other tools on human and 5 cross-species datasets in McNemar's test.

	**Human**	**Mouse**	**Bos taurus**	**C.elegans**	**Chimpanzee**	**Gorilla**
CNCI v.s. lncRNA_Mdeep	4.1×10^{-115}	0	0	0	0	0
CPAT v.s. lncRNA_Mdeep	8.6×10^{-94}	3.9×10^{-126}	3.0×10^{-3}	0	4.3×10^{-5}	9.9×10^{-12}
PLEK v.s. lncRNA_Mdeep	0	0	0	0	0	0
lncRNA-MFDL v.s. lncRNA_Mdeep	1.0×10^{-135}	3.3×10^{-256}	1.1×10^{-90}	4.7×10^{-22}	2.5×10^{-135}	3.3×10^{-6}
CPC2 v.s. lncRNA_Mdeep	0	0	1.4×10^{-54}	3.5×10^{-203}	1.0×10^{-37}	1.5×10^{-42}
lncRNAnet v.s. lncRNA_Mdeep	9.6×10^{-5}	3.0×10^{-8}	4.7×10^{-42}	5.1×10^{-27}	2.6×10^{-111}	1.9×10^{-42}
lncFinder[1] v.s. lncRNA_Mdeep	1.0×10^{-138}	0	4.9×10^{-6}	0	1.2×10^{-8}	3.8×10^{-19}
lncFinder[2] v.s. lncRNA_Mdeep	1.0×10^{-120}	3.6×10^{-139}	3.2×10^{-5}	0	4.0×10^{-3}	4.7×10^{-15}

Table S5. Performance of lncRNA_Mdeep and other model architectures on the high-quality dataset* in 10CV test

	**ACC (%)**	S_n **(%)**	S_p **(%)**	**MCC**
OFH_DNN	87.88 ± 1.78	89.81 ± 2.97	85.96 ± 2.61	0.7805 ± 0.0323
k-mer_DNN	88.40 ± 1.35	78.42 ± 2.69	98.37 ± 1.26	0.7841 ± 0.0242
One-hot_CNN	94.87 ± 0.81	95.69 ± 1.58	94.06 ± 2.67	0.8984 ± 0.0151
OFH_DNN + *k*-mer_DNN	91.10 ± 2.98	83.44 ± 6.14	98.75 ± 0.72	0.8331 ± 0.0529
k-mer_DNN + One-hot_CNN	96.35 ± 1.37	93.84 ± 3.01	98.87 ± 0.89	0.9288 ± 0.0256
OFH_DNN + One-hot_CNN	95.80 ± 0.96	95.95 ± 2.05	95.65 ± 2.33	0.9168 ± 0.0189

Decision fusion	97.03 ± 0.40	95.88 ± 0.81	98.19 ± 0.27	0.9440 ± 0.0072
lncRNA_Mdeep	97.88 ± 0.85	96.48 ± 1.94	99.28 ± 0.51	0.9583 ± 0.0163

The high-quality dataset*: we built the high-quality dataset by carefully re-filtering the lncRNA transcripts downloaded from GENCODE. First, we filtered the lncRNA transcripts based on the annotation levels in GENCODE. Level 1 indicates transcripts that have been experimentally validated, level 2 indicates transcripts that have been manually annotated, and level 3 indicates the predicted transcripts by Ensembl's automated annotation pipeline. We collected 2,499 level 1 lncRNA transcripts and 27,011 level 2 lncRNA transcripts. Then, considering the possible false annotation for manually annotated lncRNAs without 5'cap and 3'polyA signals, we filtered the level 2 lncRNA transcripts based on 5'cap and 3'polyA evidences. We found 360 lncRNA transcripts with 5'cap signals and 6,005 lncRNA transcripts with 3'polyA signals. And there are 147 lncRNA transcripts with both 5'cap and 3'polyA signals. Finally, we collected 2,646 (2,499 + 147) lncRNA transcripts and randomly selected the same number of protein-coding transcripts to build the high-quality dataset.

Table S6. The statistics of all datasets

	**Source**	**lncRNA**	**PCT***	**Total**
Human-Training	Gencode V30 [1]	23,000	23,000	46,000
Human-Testing	Gencode V30 [1]	6,000	6,000	12,000
Mouse	Gencode M20 [1]	17,885	57,992	75,877
Arabidopsis	RefSeq v94 [2]	4,081	47,135	51,216
Bos taurus	RefSeq v94 [2]	6,822	49,547	56,369
C. elegans	RefSeq v94 [2]	1,665	26,849	28,514
Chicken	RefSeq v94 [2]	5,681	34,130	39,811
Chimpanzee	RefSeq v94 [2]	10,709	59,186	69,895
Frog	RefSeq v94 [2]	2,793	32,794	35,587
Fruit fly	RefSeq v94 [2]	2,853	26,433	29,286
Gorilla	RefSeq v94 [2]	4,457	38,615	43,072
Pig	RefSeq v94 [2]	7,841	42,664	50,505
Zebrafish	RefSeq v94 [2]	5,935	43,129	49,064

PCT*: Protein Coding Transcript.

Reference:

1. Frankish, A.; Diekhans, M.; Ferreira, A.M.; Johnson, R.; Jungreis, I.; Loveland, J.; Mudge, J.M.; Sisu, C.; Wright, J.; Armstrong, J., et al. GENCODE reference annotation for the human and mouse genomes. *Nucleic Acids Res* **2019**, *47*, D766-D773, doi:10.1093/nar/gky955.
2. Pruitt, K.D.; Brown, G.R.; Hiatt, S.M.; Thibaud-Nissen, F.; Astashyn, A.; Ermolaeva, O.; Farrell, C.M.; Hart, J.; Landrum, M.J.; McGarvey, K.M., et al. RefSeq: an update on mammalian reference sequences. *Nucleic Acids Res* **2014**, *42*, D756-763, doi:10.1093/nar/gkt1114.
